# Inflammation Mediated Epileptogenesis as Possible Mechanism Underlying Ischemic Post-stroke Epilepsy

**DOI:** 10.3389/fnagi.2021.781174

**Published:** 2021-12-13

**Authors:** Anna Regina Tröscher, Joachim Gruber, Judith N. Wagner, Vincent Böhm, Anna-Sophia Wahl, Tim J. von Oertzen

**Affiliations:** ^1^Neurology I, Neuromed Campus, Kepler Universitätsklinikum, Linz, Austria; ^2^Medical Faculty, Johannes Kepler University, Linz, Austria; ^3^Brain Research Institute, University of Zurich, Zurich, Switzerland; ^4^Central Institute of Mental Health, University of Heidelberg, Mannheim, Germany

**Keywords:** neuroinflammation, ischemia, post-stroke seizures, gliosis, BBB leakage

## Abstract

Post-stroke Epilepsy (PSE) is one of the most common forms of acquired epilepsy, especially in the elderly population. As people get increasingly older, the number of stroke patients is expected to rise and concomitantly the number of people with PSE. Although many patients are affected by post-ischemic epileptogenesis, not much is known about the underlying pathomechanisms resulting in the development of chronic seizures. A common hypothesis is that persistent neuroinflammation and glial scar formation cause aberrant neuronal firing. Here, we summarize the clinical features of PSE and describe in detail the inflammatory changes after an ischemic stroke as well as the chronic changes reported in epilepsy. Moreover, we discuss alterations and disturbances in blood-brain-barrier leakage, astrogliosis, and extracellular matrix changes in both, stroke and epilepsy. In the end, we provide an overview of commonalities of inflammatory reactions and cellular processes in the post-ischemic environment and epileptic brain and discuss how these research questions should be addressed in the future.

## Introduction

Ischemic strokes are among the most common causes of death and account for a large proportion of disabilities in Western societies (Deuschl et al., 2020). Importantly, cerebrovascular diseases are one of the main reasons for acquired epilepsy in adulthood, so-called post-stroke epilepsy (PSE; Pitkänen et al., 2016). With the rising number of geriatric patients worldwide, the number of stroke patients, and hence PSE patients, can be expected to rise significantly in the next decades (Deuschl et al., 2020). Although good descriptions and reviews of current clinical knowledge of PSE are available (Pitkänen et al., 2016; Feyissa et al., 2019; Xu, 2019; Zelano et al., 2020; Galovic et al., 2021), surprisingly little is known about the underlying pathomechanism that leads to PSE. Therefore, treatment and preventive actions are limited and speculative.

Common hypotheses for the post-ischemic epileptogenesis include gliosis, chronic inflammation, angiogenesis, neurodegeneration, altered synaptic plasticity, or synaptic sprouting (Li et al., 2010; Feyissa et al., 2019). Additionally, blood-brain-barrier (BBB) leakage during stroke induces inflammation and pro-epileptogenic mechanisms (Vezzani et al., 2019). Mitochondrial dysfunction, edema, or ion gradient imbalances might contribute to stroke lesion size as well as to seizure generation. Moreover, seizure-like brain activity during ischemia also increases the infarct size and negatively impacts functional recovery (Williams and Tortella, 2002). This suggests that post-ischemic pathological changes and seizure generation are reciprocal processes which influence each other (Feyissa et al., 2019).

We here summarize clinical features of PSE and describe characteristics of neuroinflammation, blood-brain-barrier perturbations, gliosis, and changes in the extracellular matrix in stroke and epilepsy. Furthermore, we delineate similarities and possible interactions between the two pathologies and propose future directions for investigating the underlying pathomechanism in PSE in a preclinical and translational approach.

## Post-Stroke Epilepsy

Post-stroke seizures occur frequently and can be categorized into acute symptomatic (≤7 days post-stroke, ASS) and remote symptomatic seizures (>7 days post-stroke, RSS). ASS is considered to be the result of local metabolic imbalances (Feyissa et al., 2019). Although ASS drastically increases the risk for developing RSS, they do not contribute to the diagnosis of PSE. However, all patients who develop a single seizure more than 7 days after a stroke are classified as suffering from PSE as they have a 71.5% increased risk of developing further seizures within the next 10 years (Hesdorffer et al., 2009) and hence fall into the category of epilepsy according to the International League against Epilepsy (Fisher et al., 2014). Numbers of patients developing PSE vary due to different cohorts regarding the length of follow-up, stroke etiology, or varying definitions of early and RSS, but generally range from 3 to 25% (Galovic et al., 2018, 2021; Feyissa et al., 2019; Xu, 2019; Ferreira-Atuesta et al., 2021). Risk factors for PSE include strokes with cortical involvement, stroke severity, young age (below 65 years), or ASS (Galovic et al., 2018; Feyissa et al., 2019; Zelano et al., 2020; Ferreira-Atuesta et al., 2021).

Hemorrhagic strokes, which only make up about 5–10% of strokes, are more epileptogenic than ischemic ones (Caplan and Kase, 2016). ASS appears in between 2% and 4% of patients with ischemic strokes vs. 10% to 16% of patients with intracranial hemorrhages (Haapaniemi et al., 2014; Wang et al., 2017; Thevathasan et al., 2018). These numbers may be an under-estimate, as a study postulates that up to a fifth of patients showed electroencephalographic seizures in the acute phase after stroke (Bentes et al., 2017). The incidence of late-onset post-stroke seizures at a 5-year follow-up has been reported to be 9.5% for ischemic strokes and 11.8% for hemorrhagic strokes (Haapaniemi et al., 2014; Holtkamp et al., 2017).

Approximately 70% of PSE patients develop focal seizures, which occasionally generalize. The remaining 30% have bilateral tonic-clonic seizures only (Xu, 2019). PSE does not only lead to decreased quality of life, as does epilepsy in general, but it has also been linked with decreased post-stroke recovery, neurological deterioration, and poor functional outcomes (Graham et al., 2013; Baranowski, 2018; Feyissa et al., 2019). Seizures in PSE patients are often well manageable with anti-epileptic therapy (AED, anti-epileptic drugs), however, up to 40% of patients remain unresponsive to pharmacological intervention (Feyissa et al., 2019; Xu, 2019). General prophylactic AED treatment after stroke is not advisable, as this has been reported to decrease behavioral and motor recovery by inhibiting neural plasticity (Messé et al., 2009). Moreover, long-term AED usage may increase the risk of atherosclerosis, which would pose an additional risk for post-stroke patients (Tan et al., 2009). Although there are some clinical tools available to determine the risk of developing PSE after ischemic, hemorrhagic, or both stroke types (Strzelczyk et al., 2010; Haapaniemi et al., 2014; Galovic et al., 2018; Lekoubou et al., 2021), an easily measurable biomarker would ameliorate early diagnosis and facilitate preventive treatment even before the first seizure has occurred. In search of imaging biomarkers predicting the risk of poststroke seizures, a significant association between the incidence of PSE 2 years after the occurrence of hemorrhagic transformation post ischemic stroke in patients who underwent endovascular revascularization therapy was found (17.9% compared with 4.0%, *p* = 0.001; Thevathasan et al., 2018). In a recent study no impact of reperfusion techniques on the development of PSE was found (Ferreira-Atuesta et al., 2021).

Although RSS is significantly associated with stroke severity and stroke location in the middle cerebral artery territory among others (Galovic et al., 2018), no explicit information regarding more accurate stroke localization, infarct size, or concomitant cerebrovascular damages, for example microangiopathy, or loss in brain volume, is available.

Unfortunately, hardly anything is known about the pathomechanism of post-ischemic epileptogenesis on a cellular and molecular level. A better understanding of these processes would help to search for possible biomarker candidates in easily accessible compartments such as the blood and could identify patients at risk of developing PSE in a paraclinical setting.

### Neuroinflammation as Possible Mechanism Underlying PSE

**Figure 1:**
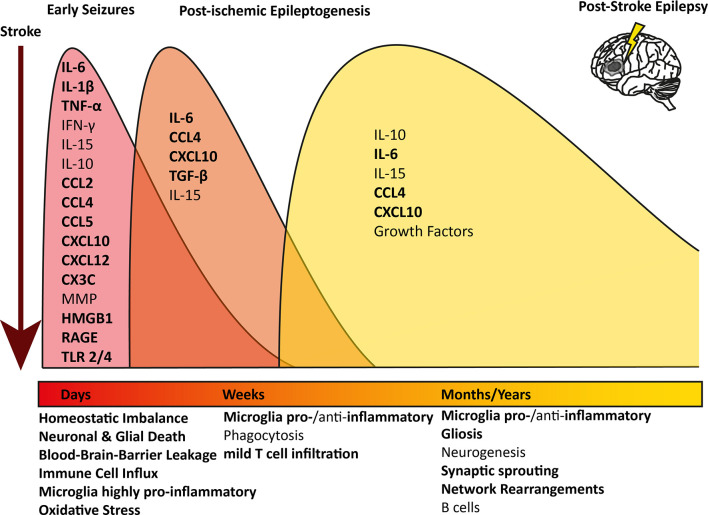
Overview of longitudinal inflammatory changes after ischemic stroke: pathophysiological mechanisms and contributing inflammatory mediators are indicated at the respective time point after an ischemic infarct. The timely progression and difference of the inflammatory profile at the different stages after stroke are indicated in different colored waves. All pathophysiological mechanisms and inflammatory mediators contributing to epilepsy too are indicated in bold.IL, Interleukin; TNF, Tumor necrosis factor; IFN, Interferon; MMP, Metalloproteinases; HMGB1,High-mobility group box 1; RAGE, receptor for advanced glycation products; TLR, Toll-like receptor.

An ischemic insult induces a plethora of processes in the brain parenchyma, such as excitotoxicity, hypoxic injury, activation of the immune system, and BBB leakage (Wimmer et al., 2018; Jayaraj et al., 2019). Interestingly, similar mechanisms have been described to induce epileptogenesis (Figure 1; Bauer et al., 2017; Vezzani et al., 2019). Especially neuroinflammation-mediated epilepsy has become a focus of attention in recent years. Several immunological mediators have been shown to lower the seizure threshold. Innate as well as adaptive neuroinflammation has repeatedly been shown in the brain parenchyma in several forms of epilepsy (Vezzani et al., 2011a; Devinsky et al., 2013; Bauer et al., 2017).

#### Neuroinflammation in Stroke

Post-ischemic inflammation occurs rapidly after the event and is characterized by microglia activation, influx of peripheral immune cells, and BBB breakdown.

In the early stage after a stroke, acute neuronal and glial cell death occurs within the necrotic area. Peripheral immune cells, above all macrophages, neutrophils, and leukocytes, infiltrate the brain and migrate into the core lesion. Microglia in the surrounding areas increase in number and polarize towards a highly pro-inflammatory phenotype, expressing markers linked to oxidative stress, phagocytosis, and antigen presentation (Zrzavy et al., 2017; Figure 1, red wave). Pro-inflammatory cytokines are elevated in post-mortem tissue of stroke patients, such as interleukin (IL) 6, IL-1β, interferon (IFN) γ, tumor necrosis factor (TNF) α and IL-15 (Doll et al., 2014; Nguyen et al., 2016; Zrzavy et al., 2017; Wimmer et al., 2018). IL-1β and TNFα levels in serum and plasma have been investigated and some studies reported an increase shortly after the stroke (Intiso et al., 2004; Sotgiu et al., 2006), whereas others did not (Tarkowski et al., 1995; Ormstad et al., 2011). However, due to the potent pro-inflammatory action of these cytokines and short half-life, a short-lived and locally restricted increase would be expected (Doll et al., 2014; Jayaraj et al., 2019). Further, many pro-inflammatory chemokines are upregulated in the brain, such as CCL1, CCL2, CCL4, CCL5, CCL22, CXCL10, and CXCL12, and some can be detected in serum or plasma (García-Berrocoso et al., 2014; Nguyen et al., 2016). On the other hand, anti-inflammatory cytokines such as IL-10 are highly upregulated in early stroke lesions and initially down-regulated in the serum, but levels increase later on (Ormstad et al., 2011; Nayak et al., 2012; Nguyen et al., 2016). Peripheral IL-6 and IL-10 levels were linked to worsened or improved stroke outcomes, respectively (Jiao et al., 2016; Nguyen et al., 2016). As microglia are polarized towards a pro-inflammatory phenotype, they can produce oxygen and nitrogen (ROS and NOS) species, which lead to BBB breakdown and act as neurotoxins (Seneş et al., 2007; Jayaraj et al., 2019). Astrocytes become hyperplasic and produce chemokines, cytokines, and astrocyte-specific proteins such as vimentin, or glial fibrillary acidic protein (GFAP), which contribute to scar formation (Wang et al., 2018; Pluta et al., 2021). Astrocytes also produce ephrin-A5, extracellular matrix molecules, and chondroit sulfate proteoglycans, which interfere with axonal sprouting, inhibit axonal growth and regeneration (Rolls et al., 2009; Overman et al., 2012; Huang et al., 2014). In the blood, increased levels of plasma lipid peroxides and thiobarbituric acids were found, a sign of ongoing oxidative stress and elevated levels of ROS and NOS (Alexandrova et al., 2003). Moreover, fraktalkine (CX3C), which can induce chemotaxis of leukocytes and microglia by binding to its receptor CX3CR1, seems to play a role in the post-ischemic outcome in an animal model, as CX3C knock-out animals had a nearly 30% reduction in infarct size and mortality (Soriano et al., 2002).

After the initial wave of inflammation, peripheral macrophages which have infiltrated the brain, together with brain-resident microglia, phagocytose debris of dead cells and shift towards either a mixed pro- and anti-inflammatory, or exclusively anti-inflammatory phenotype linked to the resolution of inflammation, removal of debris, and central nervous system (CNS) remodeling (Zrzavy et al., 2017). Pro-inflammatory mediators, such as IL-6, CXCL10, and CCL4, but also anti-inflammatory transforming growth factor (TGF) β are still significantly elevated (Nguyen et al., 2016; Jayaraj et al., 2019; Figure 1, orange wave). In post-mortem brains of patients, who died within 7 days after an ischemic infarct, astrocytes were shown to produce pro-inflammatory IL-15 *via* activating CD8+ T cells and natural killer cells (Li et al., 2017). Lymphocytes, especially CD8+ T cells, were increased in the ischemic lesion compared to healthy controls, although the number of T cells was about 10-fold lower compared to inflammatory conditions such as multiple sclerosis or encephalitis and did not show active proliferation as a sign of antigen recognition (Zrzavy et al., 2017).

In the late cystic or scar stage, an astrocytic scar has formed around the necrotic core. In this late phase after ischemic stroke, microglia reappear within the lesion core with a partly homeostatic, partly inflammatory phenotype (Zrzavy et al., 2017). Several pro- and anti-inflammatory cytokines and chemokines are still elevated and microglia produce increased levels of various growth factors, enhancing neurogenesis and plasticity within the penumbra (Nguyen et al., 2016; Jayaraj et al., 2019; Figure 1, yellow wave). B cells have been postulated to play a role in stroke recovery in the late stage. Increased numbers of regulatory B cells were shown to be beneficial, as they secret anti-inflammatory IL-10 (Offner and Hurn, 2012). On the other hand, in a mouse model for stroke, activated B cells infiltrated the brain weeks after a stroke and secreted immunoglobulin, leading to cognitive deficits. Post-stroke cognitive decline was decreased drastically upon depletion of B cells, indicating an active role of the activated B cells in the brain (Doyle et al., 2015).

#### Neuroinflammation in Epilepsy

In epilepsy, the temporal resolution of inflammatory processes is less well established. In humans, the epileptogenic processes leading to the first seizure can occur over years during which patients are free of symptoms. Most data on human patients derive from surgical resections of drug-resistant epilepsy patients, in whom epileptogenic activity has been ongoing for years. Hence, the pathological changes characterize a very late stage of the disease, and no conclusions regarding the initial events can be drawn. Results from both, human end-point analyses and animal studies, where clear time-course studies are feasible, revealed several pro-inflammatory mediators, which are involved in epileptogenesis. The most prominent one is IL-1β, which is produced in the brain of human patients with pharmaco-resistant epilepsy and was also shown to lower the seizure threshold in animals (Vezzani et al., 1999, 2000, 2011b). IL-1β leads to an altered phosphorylation of NMDA receptors, rendering them more permeable to calcium and therefore increasing neuronal firing or potentially even excitotoxicity (Viviani et al., 2003). Moreover, patients’ serum IL-1β levels decrease after surgical resection of the epileptogenic foci (Pedre et al., 2018a). IL-6 is upregulated during epileptogenesis, decreases neurogenesis, and promotes gliosis (Minami et al., 1991; Ichiyama et al., 1998; Peltola et al., 2000; Valliéres et al., 2002; Liimatainen et al., 2009; Rana and Musto, 2018). Moreover, IL-6 serum levels were reduced after surgical resection of the epileptogenic foci and were significantly reduced in epileptic patients who became seizure-free after the surgery (Pedre et al., 2018a). Further, increased levels of oxidative stress markers as a possible sign for activated, pro-inflammatory microglia, were found in the blood of epilepsy patients, which decreased after surgery (López et al., 2007; Pedre et al., 2018b). Increased levels of inducible nitric oxide synthase (iNOS) were found in post-mortem tissue of epilepsy patients (Pauletti et al., 2019; Terrone et al., 2019). TNFα can exert various pro-epileptogenic properties in the brain (Minami et al., 1991; Ichiyama et al., 1998; Arulsamy and Shaikh, 2020). It is released from activated microglia upon extracellular glutamate detection and leads to an upregulation of synapses. *In vitro*, TNFα induces an upregulation of AMPA receptors and the endocytosis of GABA receptors (Stellwagen et al., 2005). Chemokines have also been depicted to play a role in epileptogenesis. In human tissue resections CCL2, CCL3, CCL4, CCL5, and CXCL10 were highly upregulated (Wu et al., 2008; Tröscher et al., 2019). The same was found in animal models, where CCL2, CCL3, CCL4, and CCL5 have been involved in epileptic neuronal signaling (Fabene et al., 2008, 2010; Foresti et al., 2009; Xu et al., 2009; Cerri et al., 2017). Moreover, an endothelial upregulation of chemokines can lead to increased leukocyte influx, which has been shown to act pro-convulsively and induce an epileptogenic inflammatory milieu within the brain parenchyma in animals (Fabene et al., 2008, 2010; Cerri et al., 2017). In humans, T cell numbers were found to be uncorrelated with seizure frequency, making a direct link between T cells and epileptogenesis less likely (Tröscher et al., 2021). The neuronal chemokine fraktalkine (CX3CL1), which mediates neuronal-microglial interaction, has been shown to modulate GABA_A_ currents in human temporal lobe epilepsy (Roseti et al., 2013; all inflammatory changes occurring in epilepsy in Figure 1 in bold).

#### Astrogliosis and Glial Scar in Stroke and Epilepsy

The astrocytic scar formed in the late stage after stroke provides a scaffold for angiogenesis, modulates immune cells and prevents the uncontrollable spread of inflammation and cell damage to healthy areas, and maintains ion and fluid balance (Rolls et al., 2009). Moreover, astrocytes forming the scar produce nerve growth factors and brain-derived neurotrophic factors (Schwartz and Nishiyama, 1994), which facilitate survival and rewiring of surviving neurons in the penumbra (Rolls et al., 2009). Several extracellular matrix proteins have also been shown to aid axonal outgrowth (Dzyubenko et al., 2018). Although neuroregeneration and rewiring are difficult in the CNS, neuronal reorganization and repair occur post-ischemia and can be enhanced by glial-derived growth factors and stimulation (Zhang and Chopp, 2009; Wahl et al., 2014, 2017).

Astrogliosis is also a hallmark of medial temporal lobe epilepsy, where areas with or without neuronal loss can be affected (Thom, 2014; Blumcke et al., 2017). Moreover, it was shown that astrocytes can change the extracellular matrix in a TGFβ-dependent manner, leading to a breakdown of perineuronal nets around inhibitory neurons, triggering inhibition deficits (Kim et al., 2017). Disruption of the extracellular matrix leads to altered ion concentrations, osmolarity, and firing behavior in hippocampal neurons (Glykys et al., 2014). Astrocytes also play a critical role with respect to ion balancing, especially potassium buffering, and neurotransmitter homeostasis. These mechanisms have been shown to be altered in epilepsy surgical resections (Pekny et al., 2016).

#### Blood-Brain-Barrier Breakdown in Stroke and Epilepsy

Another hallmark of stroke is BBB breakdown. This is mainly mediated by proteinases such as metalloproteinases (mainly MMP2 and MMP9), which are induced by hypoxia-inducible factor (HIF) 1α or various pro-inflammatory cytokines. They directly decrease the expression of tight-junction proteins and shift their location. MMP9 levels were found to be increased after ischemic stroke and positively correlate with the severity of neurological deficits. Interestingly, MMP9 levels dropped after 72 h, except in patients with stroke progression (Brouns et al., 2011). Integrins, which under physiological conditions interact with the basement membrane to control BBB permeability, also break down, leading to edema and exacerbated inflammation (Yang and Rosenberg, 2011). In rodent stroke models, reactive oxygen species were shown to further enhance BBB leakage (Kim et al., 2001). Breakdown of the BBB leads to leakage of peripheral proteins such as albumin, which by itself acts highly pro-inflammatory within the brain parenchyma (Brouns et al., 2011; Altman et al., 2019; Yang et al., 2019).

BBB leakage also commonly occurs in epilepsy and also increases the risk of developing epilepsy after a precipitating incident (Marchi et al., 2007, 2010, 2012; van Vliet et al., 2007; Tomkins et al., 2008; Raabe et al., 2012). Peripheral proteins can induce seizures and lead to imbalances in brain homeostasis and trigger inflammation. Albumin was shown to decrease potassium buffering in a TGFβ-dependent manner (Schröder et al., 2000; Jauch et al., 2002; Cacheaux et al., 2009; Frigerio et al., 2012; Heinemann et al., 2012; Devinsky et al., 2013; Gorter et al., 2019). Moreover, seizures themselves can lead to increased BBB permeability (Marchi et al., 2007, 2012; van Vliet et al., 2007; Tomkins et al., 2008; Morin-Brureau et al., 2011; Devinsky et al., 2013; Vezzani et al., 2019). The importance of BBB leakage with respect to the development of PSE is further underlined by the fact that statins reduce the risk for hospitalization for epilepsy after stroke in a dose-dependent manner. Their effect is mainly ascribed to their anti-inflammatory and protective action on the BBB (Xu et al., 2020; Fang et al., 2021; Guo et al., 2021).

#### Alarmins in Stroke and Epilepsy

Inflammation in stroke as well as in epilepsy can be initiated, propagated, and maintained by the release of alarmins, which are mostly intracellular molecules released upon cellular stress or death. In stroke, various alarmins, such as the protein high-mobility group box 1 (HMGB1), purins, or peroxireduxins, are released and bind to various damage associated molecular pattern receptors (DAMPs), initiating an inflammatory cascade (Gülke et al., 2018). HMGB1 activates the receptor for advanced glycation products (RAGE) as well as toll-like receptor (TLR) 2 and 4, which are also activated by peroxiredoxins. Similar pathways, mediated *via* HMGB1, RAGE, and TLR4 were shown to be upregulated in epilepsy animal models and surgical resections of epilepsy patients (Zurolo et al., 2011; Paudel et al., 2019). HMGB1 was also found in the serum of stroke patients and high levels were linked to a worse outcome, indicated by higher follow-up modified Rankin scores at 1 year (Tsukagawa et al., 2017). Binding to these receptors initiates NF_K_B signaling, which induces the transcription of several highly pro-inflammatory chemokines and cytokines (Liu et al., 2017) and was shown to be activated in hippocampal resections of medial temporal lobe epilepsy patients (Crespel et al., 2002). Purins, which are released from dying cells after seizures or ischemia, can activate the inflammasome *via* Nod-like receptor (NLRP) 3, which triggers the secretion of IL1β and IL18 (Beamer et al., 2016; Gülke et al., 2018). The inflammasome or its products IL1β and IL18 have been shown to be upregulated in various epilepsies (Tröscher et al., 2019; Vezzani et al., 2019).

### Commonalities in the Inflammatory Activation Between Epilepsy and Stroke

There is a significant overlap of inflammatory mechanisms after an ischemic infarct compared to findings in epilepsy patients. Pro-inflammatory cytokines, such as IL-6, IL-1β, and TNFα have been described in brains and in the blood of both, patients after stroke and in epilepsy (Table 1; Stellwagen et al., 2005; Doll et al., 2014; Nguyen et al., 2016; Zrzavy et al., 2017; Pedre et al., 2018a; Rana and Musto, 2018; Wimmer et al., 2018; Vezzani et al., 2019). Especially the pro-epileptogenic action of IL-1β and TNFα are well understood and their upregulation after ischemia could be a possible explanation for post-ischemic epileptogenesis (Stellwagen et al., 2005; Vezzani et al., 2019). Alarmins, secreted due to neuronal damage or cell death caused by the ischemia, may augment the inflammatory reaction and activate pro-epileptogenic pathways *via* pro-inflammatory cytokines (Vezzani et al., 2011b; Gülke et al., 2018). A recent study found downregulation of calcium-binding protein B (S100B) and heat-shock protein (Hsc70) as well as an upregulation of endostatin in patients directly after stroke, who had a high probability of developing PSE in the following months (Abraira et al., 2020). S100B and Hsc70 are DAMPs linked to BBB integrity, hence lower levels could point towards perturbed BBB function (Galovic et al., 2021). In addition, T cell and monocyte-attracting chemokines are expressed in the early, intermediate, and late stages after an ischemic infarct and in epilepsy resections (Fabene et al., 2010; García-Berrocoso et al., 2014; Cerri et al., 2017). How these chemokines act pro-epileptogenically is not yet fully understood, but T cells have been shown to infiltrate the brain in both epilepsy and after an ischemic stroke, albeit in low numbers (Zrzavy et al., 2017; Tröscher et al., 2021). However, a recent study showed that the number of T cells in medial temporal lobe epilepsy does not correlate with the seizure frequency. Therefore, parenchymal T cells are unlikely to be the key driver of post-ischemic epileptogenesis (Tröscher et al., 2021). However, so far little is known about the parenchymal inflammatory milieu in PSE brains.

**Table 1:** Summary of Inflammatory changes in stroke and epilepsy.

	**Stroke**	**Epilepsy**
Cell Loss	Neuronal and Glial Cell Death	Neuronal Loss (depending on etiology)
Immune Cells Involved	Macrophages, Neutrophils, Leukocytes, Lymphocytes, Microglia	Microglia, Lymphocytes
Microglia	First pro-inflammatory, then phagocytic, produce growth factors	Chronically pro-inflammatory, ramified morphology
Cytokines	IL-6, IL-1β, IL-15, IL-10, IFN-γ, TNF-α, TGF-β	IL-6, IL-1β, TNF-α, TGF-β
Chemokines	CCL1, CCL2, CCL4, CCL5, CCL22, CXCL10, CXCL12, CX3C	CCL2, CCL3, CCL4, CCL5, CXCL10, CX3CL1
Reactive Oxygen and Nitrogen Species	Plasma lipid peroxides and thiobarbituric acid in blood	Increased in blood, iNOS in post-mortem brains
Astrocytes	Hyperplasic, produce increased vimentin, GFAP, ephrin-A5, ECM molecules, CSPGs, nerve growth factors, BDNF, scar formation	Astrogliosis, ramified morphology
Blood-Brain Barrier	BBB breakdown (acute), HIF-1α induced MMP2 and MMP9, integrin breakdown, albumin leakage	BBB leakage (chronic), imbalance in brain homeostasis, albumin leakage
Alarmins	HMGB1, purins, peroxireduxins, RAGE, TLR2 and 4, S100B and Hsc70 downregulation in patients with a high probability of PSE	HMGB1, RAGE, TLR4
Network Rearrangements	Plasticity in the penumbra, axonal outgrowth through ECM proteins, neuroregeneration and rewiring (limited), synaptic sprouting	Synaptic sprouting in hippocampus

IL, Interleukin; TNF, Tumor necrosis factor; MMP, Metalloproteinases; HMGB1, High-mobility group box 1; RAGE, receptor for advanced glycation products; TLR, Toll-like receptor.

Glial scar forming astrocytes are present around the lesion core after an ischemic infarct and are commonly found in epilepsy patients, for example in hippocampal sclerosis (Rolls et al., 2009; Robel and Sontheimer, 2015; Wang et al., 2018). Moreover, astrocytes play a key role in neurotransmitter homeostasis (Pekny et al., 2016). The glial scar and excessively produced extracellular matrix proteins around the lesion core may inhibit axonal regrowth and prevent physiological synaptic sprouting. Therefore, excessive scar formation around the lesion could potentially affect surviving neurons within the penumbra/scar area or neurons in the neighboring areas (Glykys et al., 2014; Robel and Sontheimer, 2015; Pekny et al., 2016; Wang et al., 2018). On the other hand, astroglial scars were also shown to produce neuronal growth factors and act beneficially in post-stroke rewiring (Schwartz and Nishiyama, 1994; Do Carmo Cunha et al., 2007; Rolls et al., 2009; Zhang and Chopp, 2009). In brain specimens of epilepsy patients, synaptic sprouting was also observed, where it was ascribed to inducing or facilitating seizure generation (Proper et al., 2000; Jarero-Basulto et al., 2018). Disturbances of previously functional networks have been shown by EEG recordings, where prolonged disturbed gamma oscillations were found in animal models and also correlated with stroke recovery in humans (Vecchio et al., 2019; Hazime et al., 2021).

Astrocytes play another critical role in post-ischemic parenchymal processes *via* their endfeet, constituting a part of the BBB (Alvarez et al., 2013). After ischemic infarcts, the BBB often breaks down leading to the influx of peripheral molecules and cells (Brouns et al., 2011). In epilepsy, BBB leakage is a commonly observed phenomenon and was repeatedly described as triggering or enhancing epileptic activity (Friedman, 2011). Therefore, chronic alterations in the BBB could be another mechanism by which PSE develops and how epileptic activity persists even months and years after the ischemic lesion occurred.

## Discussion and Future Directions

Although PSE is one of the most common forms of acquired epilepsy in the elderly, surprisingly little is known about the underlying pathomechanisms. One of the most common theories for post-ischemic epileptogenesis is chronic inflammation, which can occur in stroke patients but also in patients suffering from epilepsy. Many studies have investigated the inflammatory cascades occurring after stroke (for review see Wimmer et al., 2018; Jayaraj et al., 2019). In epilepsy research chronic inflammation has been delineated as a potent driver of epileptogenesis as well (Wilcox and Vezzani, 2014; Bauer et al., 2017; Vezzani et al., 2019). However, hardly anything is known about the interaction of these two pathomechanisms.

In recent years, crucial scientific work has been done to pave the way for future studies, among them establishing a sensitive clinical screening tool for patients at high risk of developing PSE (Galovic et al., 2018). This will allow for designing studies to investigate possible pathomechanisms and therapeutic approaches for PSE by pre-selecting susceptible patients. Previously, designing prospective studies on PSE was hardly realistic due to the relatively low percentage of patients developing PSE. Using the SELECT score, a patient pool with patients at high risk for PSE can be generated and followed up. First investigations on blood biomarkers in PSE have recently been published and underline the fact, that inflammatory processes might be involved in the pathomechanism of PSE (Abraira et al., 2020). Furthermore, the recently published meta-analyses on the beneficial effect of statins on the development of PSE point towards a pro-inflammatory origin of RSS (Guo et al., 2015; Xu et al., 2020; Fang et al., 2021). As clinical research on PSE is very laborious and time-consuming, translational research would be very useful to understand the basic pathomechanisms. However, generating animal models is equally difficult, as mice and rats also tend to develop seizures after stroke in relatively low numbers. Hence, high numbers of animals would be required for reliable and reproducible results. Moreover, the validity of results gained from young rodents for a human disease affecting the elderly is questionable (Reddy et al., 2017). *In vitro* approaches using human iPSCs or organotypic slices could lead to insights on basic molecular principles of post-ischemic epileptogenesis with human cells or tissues. However, most likely only combining results from all three approaches will lead to a better understanding of the underlying pathomechanism of post-ischemic PSE and thus help to identify targets for therapy and prophylaxis.

We summarized the key inflammatory mediators involved in both diseases and provided a broad overview on potential post-ischemic epileptogenic mechanisms. In the future, these questions have to be addressed in clinical as well as translational research to provide insights into the development of PSE. In animal models, the basic principles of post-ischemic inflammation and their potential to drive epileptogenesis can be studied in detail, allowing exactly timed disease course analyses and *in vivo* monitoring of neuronal activity. Moreover, in animal models confounding factors such as lesion size, age, and area of incident can easily be controlled for. In clinical research, the quest for biomarkers in easily accessible compartments, such as the blood, will be crucial. Moreover, electroencephalographic and imaging approaches can be used in human clinical research to identify key epileptogenic hubs and their specific firing properties during the latent phase of epileptogenesis. This will not only improve our basic understanding of post-ischemic brain physiology but also pave the way for potential prophylactic interventions, inhibiting epileptogenesis before the first seizure occurs.

## Author Contributions

AT and VB summarized the molecular biological aspects of stroke and epilepsy. JG and JW summarized the clinical aspects of PSE. A-SW contributed with her knowledge on stroke and stroke rehabilitation. AT wrote the manuscript. TO supervised the project. All authors contributed to the article and approved the submitted version.

## Conflict of Interest

JW reports personal fees from UCB and Boehringer Ingelheim and non-financial support from Roche, outside the submitted work; TO reports personal fees and non-financial support from Eisai Pharma GmbH Vienna, grants, personal fees, and non-financial support from UCB Pharma GmbH Vienna, non-financial support from Medtronic Austria GmbH, grants, personal fees, and non-financial support from Novartis Pharma, personal fees from Roche Pharma, personal fees from Biogen Idec Austria, personal fees from Liva Nova, personal fees from Sanofi-Aventis GmbH, grants from Grossegger & Drbal GmbH, outside the submitted work. The remaining authors declare that the research was conducted in the absence of any commercial or financial relationships that could be construed as a potential conflict of interest.

## Publisher’s Note

All claims expressed in this article are solely those of the authors and do not necessarily represent those of their affiliated organizations, or those of the publisher, the editors and the reviewers. Any product that may be evaluated in this article, or claim that may be made by its manufacturer, is not guaranteed or endorsed by the publisher.

## References

[B1] Abraira L. Santamarina E. Cazorla S. Bustamante A. Quintana M. Toledo M.. (2020). Blood biomarkers predictive of epilepsy after an acute stroke event. Epilepsia 61, 2244–2253. 10.1111/epi.16648 32857458

[B2] Alexandrova M. L. Bochev P. G. Markova V. I. Bechev B. G. Popova M. A. Danovska M. P.. (2003). Oxidative stress in the chronic phase after stroke. Redox Rep. 8, 169–176. 10.1179/135100003225001548 12935315

[B3] Altman K. Shavit-Stein E. Maggio N. (2019). Post stroke seizures and epilepsy: from proteases to maladaptive plasticity. Front. Cell. Neurosci. 13:397. 10.3389/fncel.2019.00397 31607864 PMC6755337

[B4] Alvarez J. I. Katayama T. Prat A. (2013). Glial influence on the blood brain barrier. Glia 61, 1939–1958. 10.1002/glia.22575 24123158 PMC4068281

[B5] Arulsamy A. Shaikh M. F. (2020). Tumor necrosis factor-α, the pathological key to post-traumatic epilepsy: a comprehensive systematic review. ACS Chem. Neurosci. 11, 1900–1908. 10.1021/acschemneuro.0c00301 32479057

[B6] Baranowski C. J. (2018). The quality of life of older adults with epilepsy: a systematic review. Seizure 60, 190–197. 10.1016/j.seizure.2018.06.002 30031296

[B7] Bauer J. Becker A. J. Elyaman W. Peltola J. Rüegg S. Titulaer M. J.. (2017). Innate and adaptive immunity in human epilepsies. Epilepsia 58, 57–68. 10.1111/epi.13784 28675562 PMC5535008

[B8] Beamer E. Gölöncsér F. Horváth G. Beko K. Otrokocsi L. Koványi B.. (2016). Purinergic mechanisms in neuroinflammation: an update from molecules to behavior. Neuropharmacology 104, 94–104. 10.1016/j.neuropharm.2015.09.019 26384652

[B9] Bentes C. Martins H. Peralta A. R. Casimiro C. Morgado C. Franco A. C.. (2017). Post-stroke seizures are clinically underestimated. J. Neurol. 264, 1978–1985. 10.1007/s00415-017-8586-9 28808783

[B10] Blumcke I. Spreafico R. Haaker G. Coras R. Kobow K. Bien C. G.. (2017). Histopathological findings in brain tissue obtained during epilepsy surgery. N. Engl. J. Med. 377, 1648–1656. 10.1056/NEJMoa1703784 29069555

[B11] Brouns R. Wauters A. De Surgeloose D. Mariën P. De Deyn P. P. (2011). Biochemical markers for blood-brain barrier dysfunction in acute ischemic stroke correlate with evolution and outcome. Eur. Neurol. 65, 23–31. 10.1159/000321965 21135557

[B12] Cacheaux L. P. Ivens S. David Y. Lakhter A. J. Bar-Klein G. Shapira M.. (2009). Transcriptome profiling reveals TGF-beta signaling involvement in epileptogenesis. J. Neurosci. 29, 8927–8935. 10.1523/JNEUROSCI.0430-09.2009 19605630 PMC2875073

[B51] Caplan L. R. Kase C. S. (2016). “Intracerebral hemorrhage,” in Caplan’s Stroke. A Clinical Approach, ed L. R. Caplan (Cambridge University Press), 477–510. 10.1017/CBO9781316095805.015

[B13] Cerri C. Caleo M. Bozzi Y. (2017). Chemokines as new inflammatory players in the pathogenesis of epilepsy. Epilepsy Res. 136, 77–83. 10.1016/j.eplepsyres.2017.07.016 28780154

[B14] Crespel A. Coubes P. Rousset M.-C. Brana C. Rougier A. Rondouin G.. (2002). Inflammatory reactions in human medial temporal lobe epilepsy with hippocampal sclerosis. Brain Res. 952, 159–169. 10.1016/s0006-8993(02)03050-0 12376176

[B15] Deuschl G. Beghi E. Fazekas F. Varga T. Christoforidi K. A. Sipido E.. (2020). The burden of neurological diseases in Europe: an analysis for the Global Burden of Disease Study 2017. Lancet Public Health 5, e551–e567. 10.1016/S2468-2667(20)30190-0 33007212

[B16] Devinsky O. Vezzani A. Najjar S. De Lanerolle N. C. Rogawski M. A. (2013). Glia and epilepsy: excitability and inflammation. Trends Neurosci. 36, 174–184. 10.1016/j.tins.2012.11.008 23298414

[B17] Do Carmo Cunha J. De Freitas Azevedo Levy B. De Luca B. A. De Andrade M. S. R. Gomide V. C. Chadi G. (2007). Responses of reactive astrocytes containing S100β protein and fibroblast growth factor-2 in the border and in the adjacent preserved tissue after a contusion injury of the spinal cord in rats: implications for wound repair and neuroregeneration. Wound Repair Regen. 15, 134–146. 10.1111/j.1524-475X.2006.00194.x 17244329

[B18] Doll D. N. Barr T. L. Simpkins J. W. (2014). Cytokines: their role in stroke and potential use as biomarkers and therapeutic targets. Aging Dis. 5, 294–306. 10.14336/AD.2014.0500294 25276489 PMC4173796

[B19] Doyle K. P. Quach L. N. Solé M. Axtell R. C. Nguyen T. V. V. Soler-Llavina G. J.. (2015). B-lymphocyte-mediated delayed cognitive impairment following stroke. J. Neurosci. 35, 2133–2145. 10.1523/JNEUROSCI.4098-14.2015 25653369 PMC4315838

[B20] Dzyubenko E. Manrique-Castano D. Kleinschnitz C. Faissner A. Hermann D. M. (2018). Role of immune responses for extracellular matrix remodeling in the ischemic brain. Ther. Adv. Neurol. Disord. 11:1756286418818092. 10.1177/1756286418818092 30619510 PMC6299337

[B21] Fabene P. F. Bramanti P. Constantin G. (2010). The emerging role for chemokines in epilepsy. J. Neuroimmunol. 224, 22–27. 10.1016/j.jneuroim.2010.05.016 20542576

[B22] Fabene P. F. Navarro Mora G. Martinello M. Rossi B. Merigo F. Ottoboni L.. (2008). A role for leukocyte-endothelial adhesion mechanisms in epilepsy. Nat. Med. 14, 1377–1383. 10.1038/nm.1878 19029985 PMC2710311

[B23] Fang J. Tuo M. Ouyang K. Xu Y. (2021). Statin on post-stroke epilepsy: a systematic review and meta-analysis. J. Clin. Neurosci. 83, 83–87. 10.1016/j.jocn.2020.11.023 33339690

[B24] Ferreira-Atuesta C. Döhler N. Erdélyi-Canavese B. Felbecker A. Siebel P. Scherrer N.. (2021). Seizures after ischemic stroke: a matched multicenter study. Ann. Neurol. 90, 808–820. 10.1002/ANA.26212 34505305 PMC9292028

[B25] Feyissa A. M. Hasan T. F. Meschia J. F. (2019). Stroke-related epilepsy. Eur. J. Neurol. 26:18–e3. 10.1111/ene.13813 30320425

[B26] Fisher R. S. Acevedo C. Arzimanoglou A. Bogacz A. Cross J. H. Elger C. E.. (2014). ILAE official report: a practical clinical definition of epilepsy. Epilepsia 55, 475–482. 10.1111/epi.12550 24730690

[B27] Foresti M. L. Arisi G. M. Katki K. Montañez A. Sanchez R. M. Shapiro L. A. (2009). Chemokine CCL2 and its receptor CCR2 are increased in the hippocampus following pilocarpine-induced status epilepticus. J. Neuroinflammation 6:40. 10.1186/1742-2094-6-40 20034406 PMC2804573

[B28] Friedman A. (2011). Blood-brain barrier dysfunction, status epilepticus, seizures and epilepsy: a puzzle of a chicken and egg? in Epilepsia, 52, 19–20. 10.1111/j.1528-1167.2011.03227.x PMC3234990 21967353

[B29] Frigerio F. Frasca A. Weissberg I. Parrella S. Friedman A. Vezzani A.. (2012). Long-lasting pro-ictogenic effects induced *in vivo* by rat brain exposure to serum albumin in the absence of concomitant pathology. Epilepsia 53, 1887–1897. 10.1111/j.1528-1167.2012.03666.x 22984896 PMC3651831

[B30] Galovic M. Döhler N. Erdélyi-Canavese B. Felbecker A. Siebel P. Conrad J.. (2018). Prediction of late seizures after ischaemic stroke with a novel prognostic model (the SeLECT score): a multivariable prediction model development and validation study. Lancet Neurol. 17, 143–152. 10.1016/S1474-4422(17)30404-0 29413315

[B31] Galovic M. Ferreira-Atuesta C. Abraira L. Döhler N. Sinka L. Brigo F.. (2021). Seizures and epilepsy after stroke: epidemiology, biomarkers and management. Drugs Aging 38, 285–299. 10.1007/s40266-021-00837-7 33619704 PMC8007525

[B32] García-Berrocoso T. Giralt D. Llombart V. Bustamante A. Penalba A. Flores A.. (2014). Chemokines after human ischemic stroke: from neurovascular unit to blood using protein arrays. Transl. Proteom. 3, 1–9. 10.1016/j.trprot.2014.03.001

[B33] Glykys J. Dzhala V. Egawa K. Balena T. Saponjian Y. Kuchibhotla K. V.. (2014). Local impermeant anions establish the neuronal chloride concentration. Science 343, 670–675. 10.1126/science.1245423 24503855 PMC4220679

[B34] Gorter J. A. Aronica E. van Vliet E. A. (2019). The roof is leaking and a storm is raging: repairing the blood-brain barrier in the fight against epilepsy. Epilepsy Curr. 19, 177–181. 10.1177/1535759719844750 31037960 PMC6610387

[B35] Graham N. S. N. Crichton S. Koutroumanidis M. Wolfe C. D. A. Rudd A. G. (2013). Incidence and associations of poststroke epilepsy the prospective South London stroke register. Stroke 44, 605–611. 10.1161/STROKEAHA.111.000220 23370202

[B36] Gülke E. Gelderblom M. Magnus T. (2018). Danger signals in stroke and their role on microglia activation after ischemia. Ther. Adv. Neurol. Disord. 11:1756286418774254. 10.1177/1756286418774254 29854002 PMC5968660

[B37] Guo J. Li J. Zhou M. Qin F. Zhang S. Wu B.. (2015). Statin treatment reduces the risk of poststroke seizures. Neurology 85, 701–707. 10.1212/WNL.0000000000001814 26203092

[B38] Guo Y. Zhu L. H. Zhao K. Guo X. M. Yang M. F. (2021). Statin use for the prevention of seizure and epilepsy in the patients at risk: a systematic review and meta-analysis of cohort studies. Epilepsy Res. 174:106652. 10.1016/j.eplepsyres.2021.106652 33971584

[B39] Haapaniemi E. Strbian D. Rossi C. Putaala J. Sipi T. Mustanoja S.. (2014). The CAVE score for predicting late seizures after intracerebral hemorrhage. Stroke 45, 1971–1976. 10.1161/STROKEAHA.114.004686 24876089

[B40] Hazime M. Alasoadura M. Lamtahri R. Quilichini P. Leprince J. Vaudry D.. (2021). Prolonged deficit of low gamma oscillations in the peri-infarct cortex of mice after stroke. Exp. Neurol. 341:113696. 10.1016/j.expneurol.2021.113696 33727098

[B41] Heinemann U. Kaufer D. Friedman A. (2012). Blood-brain barrier dysfunction, TGFβ signaling and astrocyte dysfunction in epilepsy. Glia 60, 1251–1257. 10.1002/glia.22311 22378298 PMC3615248

[B42] Hesdorffer D. C. Benn E. K. T. Cascino G. D. Hauser W. A. (2009). Is a first acute symptomatic seizure epilepsy? Mortality and risk for recurrent seizure. Epilepsia 50, 1102–1108. 10.1111/j.1528-1167.2008.01945.x 19374657

[B43] Holtkamp M. Beghi E. Benninger F. Kälviäinen R. Rocamora R. Christensen H. (2017). European Stroke Organisation guidelines for the management of post-stroke seizures and epilepsy. Eur. Stroke J. 2, 103–115. 10.1177/2396987317705536 31008306 PMC6453212

[B44] Huang L. Wu Z. B. ZhuGe Q. Zheng W. M. Shao B. Wang B.. (2014). Glial scar formation occurs in the human brain after ischemic stroke. Int. J. Med. Sci. 11, 344–348. 10.7150/ijms.8140 24578611 PMC3936028

[B45] Ichiyama T. Nishikawa M. Yoshitomi T. Hayashi T. Furukawa S. (1998). Tumor necrosis factor-alpha, interleukin-1 beta and interleukin-6 in cerebrospinal fluid from children with prolonged febrile seizures. Comparison with acute encephalitis/encephalopathy. Neurology 50, 407–411. 10.1212/wnl.50.2.407 9484363

[B46] Intiso D. Zarrelli M. M. Lagioia G. Di Rienzo F. Checchia De Ambrosio C. Simone P.. (2004). Tumor necrosis factor alpha serum levels and inflammatory response in acute ischemic stroke patients. Neurol. Sci. 24, 390–396. 10.1007/s10072-003-0194-z 14767684

[B47] Jarero-Basulto J. J. Gasca-Martínez Y. Rivera-Cervantes M. C. Ureña-Guerrero M. E. Feria-Velasco A. I. Beas-Zarate C. (2018). Interactions between epilepsy and plasticity. Pharmaceuticals (Basel) 11:17. 10.3390/ph11010017 29414852 PMC5874713

[B48] Jauch R. Windmüller O. Lehmann T. N. Heinemann U. Gabriel S. (2002). Effects of barium, furosemide, ouabaine and 4,4’-diisothiocyanatostilbene-2,2’-disulfonic acid (DIDS) on ionophoretically-induced changes in extracellular potassium concentration in hippocampal slices from rats and from patients with epilepsy. Brain Res. 925, 18–27. 10.1016/s0006-8993(01)03254-1 11755897

[B49] Jayaraj R. L. Azimullah S. Beiram R. Jalal F. Y. Rosenberg G. A. (2019). Neuroinflammation: friend and foe for ischemic stroke. J. Neuroinflammation 16:142. 10.1186/s12974-019-1516-2 31291966 PMC6617684

[B50] Jiao J.-T. Cheng C. Ma Y.-J. Huang J. Dai M.-C. Jiang C.. (2016). Association between inflammatory cytokines and the risk of post-stroke depression and the effect of depression on outcomes of patients with ischemic stroke in a 2-year prospective study. Exp. Ther. Med. 12, 1591–1598. 10.3892/etm.2016.3494 27588080 PMC4998048

[B52] Kim G. W. Lewén A. Copin J. C. Watson B. D. Chan P. H. (2001). The cytosolic antioxidant, copper/zinc superoxide dismutase, attenuates blood-brain barrier disruption and oxidative cellular injury after photothrombotic cortical ischemia in mice. Neuroscience 105, 1007–1018. 10.1016/s0306-4522(01)00237-8 11530238

[B53] Kim S. Y. Senatorov V. V. Morrissey C. S. Lippmann K. Vazquez O. Milikovsky D. Z.. (2017). TGFβ signaling is associated with changes in inflammatory gene expression and perineuronal net degradation around inhibitory neurons following various neurological insults. Sci. Rep. 7:7711. 10.1038/s41598-017-07394-3 28794441 PMC5550510

[B54] Lekoubou A. Debroy K. Kwegyir-Aggrey A. Bonilha L. Kengne A. Chinchilli V. (2021). Risk models to predict late-onset seizures after stroke: a systematic review. Epilepsy Behav. 121:108003. 10.1016/j.yebeh.2021.108003 34029995

[B55] Li M. Li Z. Yao Y. Jin W. N. Wood K. Liu Q.. (2017). Astrocyte-derived interleukin-15 exacerbates ischemic brain injury *via* propagation of cellular immunity. Proc. Natl. Acad. Sci. U S A 114, E396–E405. 10.1073/pnas.1612930114 27994144 PMC5255606

[B56] Li S. Overman J. J. Katsman D. Kozlov S. V. Donnelly C. J. Twiss J. L.. (2010). An age-related sprouting transcriptome provides molecular control of axonal sprouting after stroke. Nat. Neurosci. 13, 1496–1504. 10.1038/nn.2674 21057507 PMC3059556

[B57] Liimatainen S. Fallah M. Kharazmi E. Peltola M. Peltola J. (2009). Interleukin-6 levels are increased in temporal lobe epilepsy but not in extra-temporal lobe epilepsy. J. Neurol. 256, 796–802. 10.1007/s00415-009-5021-x 19252806

[B58] Liu T. Zhang L. Joo D. Sun S. C. (2017). NF-κB signaling in inflammation. Signal Transduct. Target. Ther. 2:17023. 10.1038/sigtrans.2017.23 29158945 PMC5661633

[B59] López J. González M. E. Lorigados L. Morales L. Riverón G. Bauzá J. Y. (2007). Oxidative stress markers in surgically treated patients with refractory epilepsy. Clin. Biochem. 40, 292–298. 10.1016/j.clinbiochem.2006.11.019 17291480

[B60] Marchi N. Angelov L. Masaryk T. Fazio V. Granata T. Hernandez N.. (2007). Seizure-promoting effect of blood-brain barrier disruption. Epilepsia 48, 732–742. 10.1111/j.1528-1167.2007.00988.x 17319915 PMC4135474

[B61] Marchi N. Granata T. Ghosh C. Janigro D. (2012). Blood-brain barrier dysfunction and epilepsy: Pathophysiologic role and therapeutic approaches. Epilepsia 53, 1877–1886. 10.1111/j.1528-1167.2012.03637.x 22905812 PMC4842020

[B62] Marchi N. Teng Q. Ghosh C. Fan Q. Nguyen M. T. Desai N. K.. (2010). Blood-brain barrier damage, but not parenchymal white blood cells, is a hallmark of seizure activity. Brain Res. 1353, 176–186. 10.1016/j.brainres.2010.06.051 20599815 PMC2933328

[B63] Messé S. R. Sansing L. H. Cucchiara B. L. Herman S. T. Lyden P. D. Kasner S. E. (2009). Prophylactic antiepileptic drug use is associated with poor outcome following ICH. Neurocrit. Care 11, 38–44. 10.1007/s12028-009-9207-y 19319701

[B64] Minami M. Kuraishi Y. Satoh M. (1991). Effects of kainic acid on messenger RNA levels of IL-1 beta, IL-6, TNF alpha and LIF in the rat brain. Biochem. Biophys. Res. Commun. 176, 593–598. 10.1016/s0006-291x(05)80225-6 1709015

[B65] Morin-Brureau M. Lebrun A. Rousset M. C. Fagni L. Bockaert J. de Bock F.. (2011). Epileptiform activity induces vascular remodeling and zonula occludens 1 downregulation in organotypic hippocampal cultures: Role of VEGF signaling pathways. J. Neurosci. 31, 10677–10688. 10.1523/JNEUROSCI.5692-10.2011 21775611 PMC6622643

[B66] Nayak A. R. Kashyap R. S. Kabra D. Purohit H. J. Taori G. M. Daginawala H. F. (2012). Time course of inflammatory cytokines in acute ischemic stroke patients and their relation to inter-alfa trypsin inhibitor heavy chain 4 and outcome. Ann. Indian Acad. Neurol. 15, 181–185. 10.4103/0972-2327.99707 22919189 PMC3424794

[B67] Nguyen T. V. V. Frye J. B. Zbesko J. C. Stepanovic K. Hayes M. Urzua A.. (2016). Multiplex immunoassay characterization and species comparison of inflammation in acute and non-acute ischemic infarcts in human and mouse brain tissue. Acta Neuropathol. Commun. 4:100. 10.1186/s40478-016-0371-y 27600707 PMC5011964

[B68] Offner H. Hurn P. D. (2012). A novel hypothesis: regulatory B lymphocytes shape outcome from experimental stroke. Transl. Stroke Res. 3, 324–330. 10.1007/s12975-012-0187-4 23175646 PMC3501272

[B69] Ormstad H. Aass H. C. D. Lund-Sørensen N. Amthor K. F. Sandvik L. (2011). Serum levels of cytokines and C-reactive protein in acute ischemic stroke patients and their relationship to stroke lateralization, type and infarct volume. J. Neurol. 258, 677–685. 10.1007/s00415-011-6006-0 21424610 PMC3065641

[B70] Overman J. J. Clarkson A. N. Wanner I. B. Overman W. T. Eckstein I. Maguire J. L.. (2012). A role for ephrin-A5 in axonal sprouting, recovery and activity-dependent plasticity after stroke. Proc. Natl. Acad. Sci. U S A 109, E2230–E2239. 10.1073/pnas.1204386109 22837401 PMC3421211

[B71] Paudel Y. Semple B. Jones N. Othman I. Shaikh M. (2019). High mobility group box 1 (HMGB1) as a novel frontier in epileptogenesis: from pathogenesis to therapeutic approaches. J. Neurochem. 151, 542–557. 10.1111/jnc.14663 30644560

[B72] Pauletti A. Terrone G. Shekh-Ahmad T. Salamone A. Ravizza T. Rizzi M.. (2019). Targeting oxidative stress improves disease outcomes in a rat model of acquired epilepsy. Brain 142:e39. 10.1093/brain/awz130 31145451 PMC6598637

[B73] Pedre L. L. Chacón L. M. M. Fuentes N. P. De Los Robinson Agramonte M. A. Sánchez T. S. Cruz-Xenes R. M.. (2018a). Follow-up of peripheral IL-1β and IL-6 and relation with apoptotic death in drug-resistant temporal lobe epilepsy patients submitted to surgery. Behav. Sci. (Basel) 8:21. 10.3390/bs8020021 29401729 PMC5836004

[B74] Pedre L. L. Gallardo J. M. Chacón L. M. M. García A. V. Flores-Mendoza M. Neri-Gómez T.. (2018b). Oxidative stress in patients with drug resistant partial complex seizure. Behav. Sci. (Basel) 8:59. 10.3390/bs8060059 29890748 PMC6027168

[B75] Pekny M. Pekna M. Messing A. Steinhäuser C. Lee J. M. Parpura V.. (2016). Astrocytes: a central element in neurological diseases. Acta Neuropathol. 131, 323–345. 10.1007/s00401-015-1513-1 26671410

[B76] Peltola J. Palmio J. Korhonen L. Suhonen J. Miettinen A. Hurme M.. (2000). Interleukin-6 and interleukin-1 receptor antagonist in cerebrospinal fluid from patients with recent tonic-clonic seizures. Epilepsy Res. 41, 205–211. 10.1016/s0920-1211(00)00140-6 10962211

[B77] Pitkänen A. Roivainen R. Lukasiuk K. (2016). Development of epilepsy after ischaemic stroke. Lancet Neurol. 15, 185–197. 10.1016/S1474-4422(15)00248-3 26597090

[B78] Pluta R. Januszewski S. Czuczwar S. J. (2021). Neuroinflammation in post-ischemic neurodegeneration of the brain: friend, foe, or both? Int. J. Mol. Sci. 22:4405. 10.3390/ijms22094405 33922467 PMC8122836

[B79] Proper E. A. Oestreicher A. B. Jansen G. H. Veelen C. W. M. v. van Rijen P. C. Gispen W. H.. (2000). Immunohistochemical characterization of mossy fibre sprouting in the hippocampus of patients with pharmaco-resistant temporal lobe epilepsy. Brain 123, 19–30. 10.1093/brain/123.1.19 10611117

[B80] Raabe A. Schmitz A. K. Pernhorst K. Grote A. Von Der Brelie C. Urbach H.. (2012). Cliniconeuropathologic correlations show astroglial albumin storage as a common factor in epileptogenic vascular lesions. Epilepsia 53, 539–548. 10.1111/j.1528-1167.2012.03405.x 22372630 PMC3669690

[B81] Rana A. Musto A. E. (2018). The role of inflammation in the development of epilepsy. J. Neuroinflammation 15:144. 10.1186/s12974-018-1192-7 29764485 PMC5952578

[B82] Reddy D. S. Bhimani A. Kuruba R. Park M. J. Sohrabji F. (2017). Prospects of modeling poststroke epileptogenesis. J. Neurosci. Res. 95, 1000–1016. 10.1002/jnr.23836 27452210 PMC5266751

[B83] Robel S. Sontheimer H. (2015). Glia as drivers of abnormal neuronal activity. Nat. Neurosci. 19, 28–33. 10.1038/nn.4184 PMC4966160 26713746

[B84] Rolls A. Shechter R. Schwartz M. (2009). The bright side of the glial scar in CNS repair. Nat. Rev. Neurosci. 10, 235–241. 10.1038/nrn2591 19229242

[B85] Roseti C. Fucile S. Lauro C. Martinello K. Bertollini C. Esposito V.. (2013). Fractalkine/CX3CL1 modulates GABAA currents in human temporal lobe epilepsy. Epilepsia 54, 1834–1844. 10.1111/epi.12354 24032743

[B86] Schröder W. Hinterkeuser S. Seifert G. Schramm J. Jabs R. Wilkin G. P.. (2000). Functional and molecular properties of human astrocytes in acute hippocampal slices obtained from patients with temporal lobe epilepsy. Epilepsia 41, S181–S184. 10.1111/j.1528-1157.2000.tb01578.x 10999541

[B87] Schwartz J. P. Nishiyama N. (1994). Neurotrophic factor gene expression in astrocytes during development and following injury. Brain Res. Bull. 35, 403–407. 10.1016/0361-9230(94)90151-1 7532097

[B88] Seneş M. Kazan N. Coşkun O. Zengi O. Inan L. Yücel D. (2007). Oxidative and nitrosative stress in acute ischaemic stroke. Ann. Clin. Biochem. 44, 43–47. 10.1016/S2665-9913(21)00315-5 17270091

[B89] Soriano S. G. Amaravadi L. S. Wang Y. F. Zhou H. Yu G. X. Tonra J. R.. (2002). Mice deficient in fractalkine are less susceptible to cerebral ischemia-reperfusion injury. J. Neuroimmunol. 125, 59–65. 10.1016/s0165-5728(02)00033-4 11960641

[B90] Sotgiu S. Zanda B. Marchetti B. Fois M. L. Arru G. Pes G. M.. (2006). Inflammatory biomarkers in blood of patients with acute brain ischemia. Eur. J. Neurol. 13, 505–513. 10.1111/j.1468-1331.2006.01280.x 16722977

[B91] Stellwagen D. Beattie E. C. Seo J. Y. Malenka R. C. (2005). Differential regulation of AMPA receptor and GABA receptor trafficking by tumor necrosis factor-α. J. Neurosci. 25, 3219–3228. 10.1523/JNEUROSCI.4486-04.2005 15788779 PMC6725093

[B92] Strzelczyk A. Haag A. Raupach H. Herrendorf G. Hamer H. M. Rosenow F. (2010). Prospective evaluation of a post-stroke epilepsy risk scale. J. Neurol. 257, 1322–1326. 10.1007/s00415-010-5520-9 20309571

[B93] Tan T.-Y. Lu C.-H. Chuang H.-Y. Lin T.-K. Liou C.-W. Chang W.-N.. (2009). Long-term antiepileptic drug therapy contributes to the acceleration of atherosclerosis. Epilepsia 50, 1579–1586. 10.1111/j.1528-1167.2009.02024.x 19292757

[B94] Tarkowski E. Rosengren L. Blomstrand C. Wikkelso C. Jensen C. Ekholm S.. (1995). Early intrathecal production of interleukin-6 predicts the size of brain lesion in stroke. Stroke 26, 1393–1398. 10.1161/01.str.26.8.1393 7631343

[B95] Terrone G. Balosso S. Pauletti A. Ravizza T. Vezzani A. (2019). Inflammation and reactive oxygen species as disease modifiers in epilepsy. Neuropharmacology 167:107742. 10.1016/j.neuropharm.2019.107742 31421074

[B96] Thevathasan A. Naylor J. Churilov L. Mitchell P. J. Dowling R. J. Yan B.. (2018). Association between hemorrhagic transformation after endovascular therapy and poststroke seizures. Epilepsia 59, 403–409. 10.1111/epi.13982 29288487

[B97] Thom M. (2014). Review: hippocampal sclerosis in epilepsy: a neuropathology review. Neuropathol. Appl. Neurobiol. 40, 520–543. 10.1111/nan.12150 24762203 PMC4265206

[B98] Tomkins O. Shelef I. Kaizerman I. Eliushin A. Afawi Z. Misk A.. (2008). Blood-brain barrier disruption in post-traumatic epilepsy. J. Neurol. Neurosurg. Psychiatry 79, 774–777. 10.1136/jnnp.2007.126425 17991703

[B99] Tröscher A. R. Sakaraki E. Mair K. M. Köck U. Racz A. Borger V.. (2021). T cell numbers correlate with neuronal loss rather than with seizure activity in medial temporal lobe epilepsy. Epilepsia 62, 1343–1353. 10.1111/epi.16914 33954995

[B100] Tröscher A. R. Wimmer I. Quemada-Garrido L. Köck U. Gessl D. Verberk S. G. S.. (2019). Microglial nodules provide the environment for pathogenic T cells in human encephalitis. Acta Neuropathol. 137, 619–635. 10.1007/s00401-019-01958-5 30663001 PMC6426829

[B101] Tsukagawa T. Katsumata R. Fujita M. Yasui K. Akhoon C. Ono K.. (2017). Elevated serum high-mobility group box-1 protein level is associated with poor functional outcome in ischemic stroke. J. Stroke Cerebrovasc. Dis. 26, 2404–2411. 10.1016/j.jstrokecerebrovasdis.2017.05.033 28645523

[B102] Valliéres L. Campbell I. L. Gage F. H. Sawchenko P. E. (2002). Reduced hippocampal neurogenesis in adult transgenic mice with chronic astrocytic production of interleukin-6. J. Neurosci. 22, 486–492. 10.1523/JNEUROSCI.22-02-00486.2002 11784794 PMC6758670

[B103] van Vliet E. A. Araújo S. D. C. Redeker S. van Schaik R. Aronica E. Gorter J. A.. (2007). Blood-brain barrier leakage may lead to progression of temporal lobe epilepsy. Brain 130, 521–534. 10.1093/brain/awl318 17124188

[B104] Vecchio F. Tomino C. Miraglia F. Iodice F. Erra C. Di Iorio R.. (2019). Cortical connectivity from EEG data in acute stroke: A study *via* graph theory as a potential biomarker for functional recovery. Int. J. Psychophysiol. 146, 133–138. 10.1016/j.ijpsycho.2019.09.012 31648028

[B106] Vezzani A. Balosso S. Ravizza T. (2019). Neuroinflammatory pathways as treatment targets and biomarkers in epilepsy. Nat. Rev. Neurol. 15, 459–472. 10.1038/s41582-019-0217-x 31263255

[B105] Vezzani A. Conti M. De Luigi A. Ravizza T. Moneta D. Marchesi F.. (1999). Interleukin-1beta immunoreactivity and microglia are enhanced in the rat hippocampus by focal kainate application: functional evidence for enhancement of electrographic seizures. J. Neurosci. 19, 5054–5065. 10.1523/JNEUROSCI.19-12-05054.1999 10366638 PMC6782637

[B107] Vezzani A. French J. Bartfai T. Baram T. Z. (2011a). The role of inflammation in epilepsy. Nat. Rev. Neurol. 7, 31–40. 10.1038/nrneurol.2010.178 21135885 PMC3378051

[B108] Vezzani A. Maroso M. Balosso S. Sanchez M. A. Bartfai T. (2011b). IL-1 receptor/Toll-like receptor signaling in infection, inflammation, stress and neurodegeneration couples hyperexcitability and seizures. Brain Behav. Immun. 25, 1281–1289. 10.1016/j.bbi.2011.03.018 21473909

[B109] Vezzani A. Moneta D. Conti M. Richichi C. Ravizza T. De Luigi A.. (2000). Powerful anticonvulsant action of IL-1 receptor antagonist on intracerebral injection and astrocytic overexpression in mice. Proc. Natl. Acad. Sci. U S A 97, 11534–11539. 10.1073/pnas.190206797 11016948 PMC17235

[B110] Viviani B. Bartesaghi S. Gardoni F. Vezzani A. Behrens M. M. Bartfai T.. (2003). Interleukin-1β enhances NMDA receptor-mediated intracellular calcium increase through activation of the Src family of kinases. J. Neurosci. 23, 8692–8700. 10.1523/JNEUROSCI.23-25-08692.2003 14507968 PMC6740426

[B111] Wahl A. S. Büchler U. Brändli A. Brattoli B. Musall S. Kasper H.. (2017). Optogenetically stimulating intact rat corticospinal tract post-stroke restores motor control through regionalized functional circuit formation. Nat. Commun. 8:1187. 10.1038/s41467-017-01090-6 29084962 PMC5662731

[B112] Wahl A. S. Omlor W. Rubio J. C. Chen J. L. Zheng H. Schröter A.. (2014). Asynchronous therapy restores motor control by rewiring of the rat corticospinal tract after stroke. Science 344, 1250–1255. 10.1126/science.1253050 24926013

[B113] Wang H. Song G. Chuang H. Chiu C. Abdelmaksoud A. Ye Y.. (2018). Portrait of glial scar in neurological diseases. Int. J. Immunopathol. Pharmacol. 31:2058738418801406. 10.1177/2058738418801406 30309271 PMC6187421

[B114] Wang J. Z. Vyas M. V. Saposnik G. Burneo J. G. (2017). Incidence and management of seizures after ischemic stroke. Neurology 89, 1220–1228. 10.1212/WNL.0000000000004407 28835405

[B115] Wilcox K. S. Vezzani A. (2014). Does brain inflammation mediate pathological outcomes in epilepsy? Adv. Exp. Med. Biol. 813, 169–183. 10.1007/978-94-017-8914-1_14 25012376 PMC4867105

[B116] Williams A. J. Tortella F. C. (2002). Neuroprotective effects of the sodium channel blocker RS100642 and attenuation of ischemia-induced brain seizures in the rat. Brain Res. 932, 45–55. 10.1016/s0006-8993(02)02275-8 11911860

[B117] Wimmer I. Zrzavy T. Lassmann H. (2018). Neuroinflammatory responses in experimental and human stroke lesions. J. Neuroimmunol. 323, 10–18. 10.1016/j.jneuroim.2018.07.003 30196821

[B118] Wu Y. Wang X. Mo X. Xi Z. Xiao F. Li J.. (2008). Expression of monocyte chemoattractant protein-1 in brain tissue of patients with intractable epilepsy. Clin. Neuropathol. 27, 55–63. 10.5414/npp27055 18402383

[B120] Xu M. Y. (2019). Poststroke seizure: optimising its management. Stroke Vasc. Neurol. 4, 48–56. 10.1136/svn-2018-000175 31105979 PMC6475084

[B119] Xu J. H. Long L. Tang Y. C. Zhang J. T. Hu H. T. Tang F. R. (2009). CCR3, CCR2A and macrophage inflammatory protein (MIP)-1α, monocyte chemotactic protein-1 (MCP-1) in the mouse hippocampus during and after pilocarpine-induced status epilepticus (PISE). Neuropathol. Appl. Neurobiol. 35, 496–514. 10.1111/j.1365-2990.2009.01022.x 19490431

[B121] Xu T. Wang Y. Yuan J. Chen Y. Luo H. (2020). Statin use and the risk of post-stroke seizures: a meta-analysis. Seizure 83, 63–69. 10.1016/j.seizure.2020.10.004 33096458

[B122] Yang C. Hawkins K. E. Doré S. Candelario-Jalil E. (2019). Neuroinflammatory mechanisms of blood-brain barrier damage in ischemic stroke. Am. J. Physiol. Cell Physiol. 316, C135–C153. 10.1152/ajpcell.00136.2018 30379577 PMC6397344

[B123] Yang Y. Rosenberg G. A. (2011). Blood-brain barrier breakdown in acute and chronic cerebrovascular disease. Stroke 42, 3323–3328. 10.1161/STROKEAHA.110.608257 21940972 PMC3584169

[B124] Zelano J. Holtkamp M. Agarwal N. Lattanzi S. Trinka E. Brigo F. (2020). How to diagnose and treat post-stroke seizures and epilepsy. Epileptic Disord. 22, 252–263. 10.1684/epd.2020.1159 32597766

[B125] Zhang Z. G. Chopp M. (2009). Neurorestorative therapies for stroke: underlying mechanisms and translation to the clinic. Lancet Neurol. 8, 491–500. 10.1016/S1474-4422(09)70061-4 19375666 PMC2727708

[B126] Zrzavy T. Machado-Santos J. Christine S. Baumgartner C. Weiner H. L. Butovsky O.. (2017). Dominant role of microglial and macrophage innate immune responses in human ischemic infarcts. Brain Pathol. 28, 791–805. 10.1111/bpa.12583 29222823 PMC6334527

[B127] Zurolo E. Iyer A. Maroso M. Carbonell C. Anink J. J. Ravizza T.. (2011). Activation of toll-like receptor, RAGE and HMGB1 signalling in malformations of cortical development. Brain 134, 1015–1032. 10.1093/brain/awr032 21414994

